# Simultaneous Immunization with Omp25 and L7/L12 Provides Protection against Brucellosis in Mice

**DOI:** 10.3390/pathogens9020152

**Published:** 2020-02-24

**Authors:** Sonal Gupta, Surender Mohan, Vikas Kumar Somani, Somya Aggarwal, Rakesh Bhatnagar

**Affiliations:** 1 Laboratory of Molecular Biology and Genetic Engineering, School of Biotechnology, Jawaharlal Nehru University, New Delhi 110067, India; sonalmole@gmail.com (S.G.); mohan.surender@gmail.com (S.M.); vikass@wustl.edu (V.K.S.); s.aggarwal@wustl.edu (S.A.); 2 Department of Oncology, Washington University School of Medicine, St. Louis, MO 63110, USA; 3 Department of Molecular Microbiology, Washington University School of Medicine, St. Louis, MO 63110, USA; 4 Banaras Hindu University, Varanasi, Uttar Pradesh 221005, India

**Keywords:** Recombinant vaccine, divalent vaccine, brucellosis, Omp25, L7/L12, *Brucella abortus* 544

## Abstract

Currently used *Brucella* vaccines, *Brucella abortus* strain 19 and RB51, comprises of live attenuated *Brucella* strains and prevent infection in animals. However, these vaccines pose potential risks to recipient animals such as attenuation reversal and virulence in susceptible hosts on administration. In this context, recombinant subunit vaccines emerge as a safe and competent alternative in combating the disease. In this study, we formulated a divalent recombinant vaccine consisting of Omp25 and L7/L12 of *B. abortus* and evaluated vaccine potential individually as well as in combination. Sera obtained from divalent vaccine (Omp25+L7/L12) immunized mice group exhibited enhanced IgG titers against both components and indicated specificity upon immunoblotting reiterating its authenticity. Further, the IgG1/IgG2a ratio obtained against each antigen predicted a predominant Th2 immune response in the Omp25+L7/L12 immunized mice group. Upon infection with virulent *B. abortus* 544, Omp25+L7/L12 infected mice exhibited superior Log10 protection compared to individual vaccines. Consequently, this study recommends that simultaneous immunization of Omp25 and L7/L12 as a divalent vaccine complements and triggers a Th2 mediated immune response in mice competent of providing protection against brucellosis.

## 1. Introduction

Brucellosis, one of the major bacterial zoonoses across the globe, is caused by members of the *Brucella* genus, (*B. abortus*, *B. melitensis*, *B. suis* and *B. canis*). It is still considered one of the seven "neglected zoonoses" worldwide, in spite of a huge public health burden in many countries with low income [1]. The transmission of brucellosis in humans occurs through coincidental exposure to pathogenic bacterium from infected animals or animal products [2,3,4,5,6]. Domestic animals affected by brucellosis are more prone to abortions whereas human brucellosis leads to debilitating symptoms such as recurrent fevers, spondylitis, joint pains, and osteomyelitis [7,8,9]. The successful vaccines available commercially against brucellosis are Strain 19 and RB51 [10]. Strain 19 is an attenuated *B. abortus* strain with smooth morphology that has the capability to induce antibody responses and protect cattle against brucellosis. *B. abortus* RB51 is a rifampicin-resistant strain with rough morphology which provides protection against infection and abortion [11]. But there are several disadvantages associated with these vaccines such as occurrence of abortions in pregnant cows, restriction on age of vaccination, and reversion of vaccine strain back to pathogenic strain upon administration [12]. Further, antibodies are directed majorly against the lipopolysaccharide O-side chain during natural infection or S19 immunization which causes obstruction during a brucellosis diagnostic test. Therefore, development of an effective and safe vaccine is required.

In this context, recombinant protein-based subunit vaccines have an advantage over traditional live-attenuated vaccines as being protective and safe for human administration [13,14,15]. Recombinant subunit vaccines rely on specific parts of the pathogenic microorganism such as proteins or capsular polysaccharides containing protective epitopes to result in a protective immune response. Since these vaccines cannot replicate in the host, they are not pathogenic on administration [16]. Numerous intracellular components and surface proteins from bacteria have been researched as potential protective antigens against *Brucella* infection, such as L7/L12 [17], Omp19, Omp31 [18], BLS [19], BP26 [15] and Omp25 [20,21], and some have shown to be effective in providing significant protection against *Brucella* infection [15,17,20,21]. Although recombinant subunit vaccines offer no residual virulence but these require administration of multiple boosters along with providing lower levels of protection [22]. Further, when these *Brucella* protective antigens were administered in combination, the induced immune responses were superior in clearing intracellular *Brucella* as compared to their univalent counterpart [14,15,18,23,24,25,26,27]. Earlier studies have suggested that Omp25 and L7/L12 can serve as efficient protective antigens by inducing strong humoral and cellular immune response [17,20,28]. Outer membrane protein 25 (Omp25) of *Brucella* species has been identified as a potential antigen [20,28] capable of inhibiting TNF-α production [21]. Importantly, *omp25* gene is highly conserved in multiple *Brucella* species, strains, and biovars [8]. In addition, L7/L12 is a conserved protein which is found to be immunogenic and a stimulant of Th1 type CD4^+^ cellular response in mice [17], making both these antigens relevant as components for divalent vaccine formulation against brucellosis. Further, many protective antigens from *Brucella* have been classified as specific and novel diagnostic target as compared to LPS based conventional tests [29,30]. 

Aluminum hydroxide (Alum) has been validated as an economical and safe adjuvant by U.S. Food and Drug administration for veterinary and human use. Aluminum salts form a short-term depot at the site of injection and slowly release antigen to the body’s immune system [31,32]. In this study, we co-immunized Omp25 and L7/L12 of *Brucella abortus* using alum as an adjuvant. Elicited antibody titers and antibody subtype profile were analyzed when administered as a divalent vaccine candidate in BALB/c mice. Further, the protective efficacy of individual proteins and the divalent vaccine candidate against virulent *B. abortus* 544 challenge were determined.

## 2. Results

### 2.1. Expression and Purification of Recombinant Proteins rOmp25 and rL7/L12 

**Figure 1:**
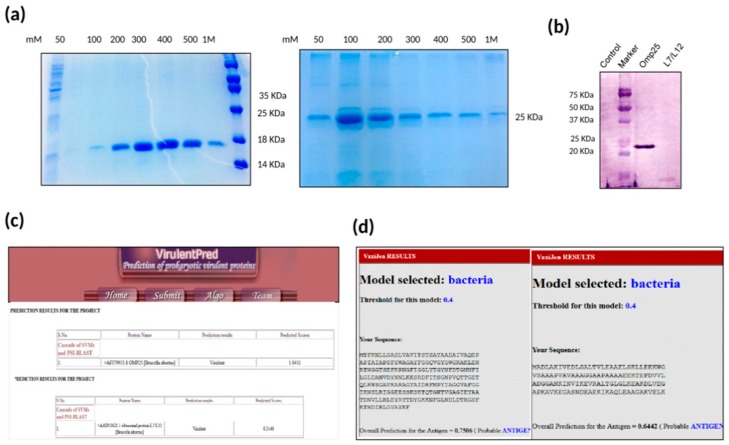
(**a**) Purification of rL7/L12 and rOmp25: SDS-PAGE gel stained with coomassie blue stain showing purification of rL7/L12 and rOmp25 recombinant proteins corresponding to 17 KDa and 25 KDa, respectively. (**b**) Immunoblotting with polyclonal sera of mice immunized with divalent vaccine (Omp25+L7/L12): The reactivity of purified proteins was confirmed by immunoblotting using anti-Omp25+L7/L12 mice serum. Negative control (lane 1; *E. coli* BL21 (DE3) cells with pET28a only), marker (lane 2) Precision Plus Protein™ Dual Color Standards, BIORAD #1610374, rOmp25 (lane 3), and rL7/L12 (lane 4). (**c**) Prediction of virulent proteins in a bacterium using VirulentPred: The sequences for *Brucella abortus* protein, Omp25 and L7/L12, have been submitted as input and their predicted scores have been calculated using VirulentPred software. (**d**) Prediction of vaccine antigens using VaxiJen: The sequences for *Brucella abortus* protein, Omp25 and L7/L12, have been submitted as input and the probability of these proteins being vaccine antigens has been predicted using VaxiJen.

The recombinant proteins rOmp25 and rL7/L12 were expressed in *E. coli* C43 cells and *E. coli* BL21 (DE3) cells respectively and purified using Ni-NTA chromatography. Further, the size of the expressed recombinant proteins was verified using 12% SDS-PAGE electrophoresis (Figure 1a). The immunoreactivity of purified proteins was confirmed by immunoblotting using (anti-Omp25+ L7/L12) mice serum signifying that serum from mice immunized with Omp25+L7/L12 consisted of antibodies specifically against its component proteins rOmp25 and rL7/L12 (Figure 1b).

Further, the virulence potential of rOmp25 and rL7/L12 was analyzed using bioinformatics analysis using VirulentPred and VaxiJen. VirulentPred is a tool used for prediction of virulent protein sequences in bacteria based on bi-layer cascade support vector machine (SVM) [33]. The SVM classifiers in this tool were trained and optimized using individual protein sequence features such as their amino acid and dipeptide composition along with position-specific iterated blast (PSI-BLAST). This tool distinguishes virulent proteins from non-virulent bacterial proteins with an accuracy of 81.8% [33]. On the basis of VirulentPred, rOmp25 and rL7/L12 were concluded to be virulent with predicted scores of 1.0411 and 0.2440, respectively (Figure 1c). In addition, we used the VaxiJen tool [34], which uses an alignment-independent approach for prediction of protective antigens. The antigen classification is purely based on the physiochemical properties of proteins without applying sequence alignment [34], and depends on auto cross covariance (ACC) transformation of protein sequences into uniform vectors of amino acid properties. VaxiJen results categorized Omp25 and L7/L12 as vaccine antigens with predicted scores of 0.7506 and 0.6442, respectively, at the threshold value of 0.4 (Figure 1d).

### 2.2. Determination of IgG Antibody Titre upon Divalent Vaccine Immunization 

**Figure 2:**
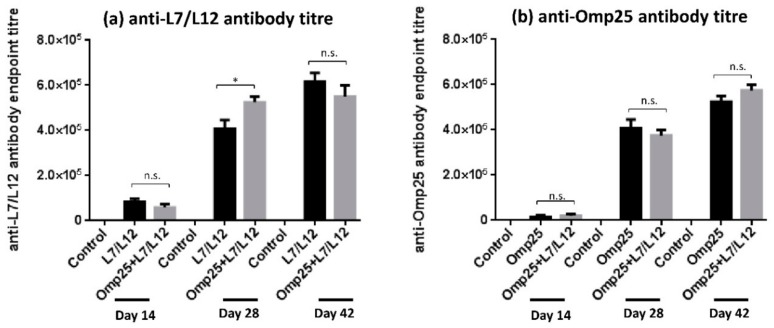
IgG antibody response elicited after immunization with L7/L12, Omp25, and divalent vaccine (Omp25+L7/L12): The mice were immunized with proteins rOmp25, rL7/L12 and rOmp25+rL7/L12 followed by isolation of serum samples from tail veins on day 14, 28, and 42. Estimation of IgG antibody end point titer was done through Enzyme linked immunosorbent assay (ELISA) and data is plotted as (mean ± SD).

To assess the levels of IgG antibody titer generated in each of the immunized mice groups, sera was collected at day 28 and 42 post-priming and levels were estimated using Enzyme linked immunosorbent assay (ELISA). Our results revealed that immunization with Omp25+L7/L12 supported a robust anti-L7/L12 IgG response that was detectable at day 28 and remained stable until day 42. At day 28, anti-L7/L12 antibodies were observed to be higher in Omp25+L7/L12 mice compared to L7/L12 only immunized mice (*p* < 0.05; Figure 2a). At day 42, anti-L7/L12 levels were found to be similar in both L7/L12 and Omp25+L7/L12 immunized mice (approximately 6 × 10^5^ in both). Immunization with divalent vaccine elicited a vigorous anti-Omp25 IgG response as well. Antibody levels were observed to be similar in Omp25+L7/L12 and Omp25 only immunized mice at day 28 and day 42 (Figure 2b). Therefore, the antigen alone vaccinated group generated antibodies only against a single antigen, whereas mice immunized with divalent vaccine (Omp25+L7/L12) produced antibodies against both components (rOmp25 and rL7/L12) in a cumulative manner, indicating that co-immunization of two proteins didn’t hinder the immune response and supported generation of antibodies against its individual components.

### 2.3. Evaluation of IgG Isotype Levels upon Divalent Vaccine Immunization

**Figure 3:**
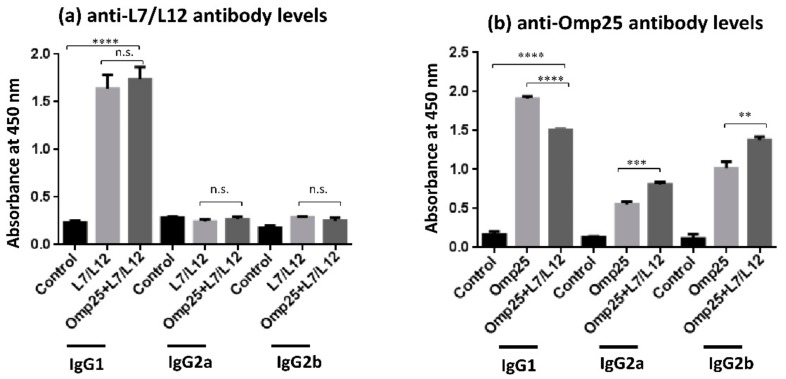
IgG antibody isotypes (IgG1, IgG2a, and IgG2b) elicited after immunization with rL7/L12, rOmp25, and divalent vaccine (Omp25+L7/L12): The recombinant *B. abortus* proteins rL7/L12, rOmp25, and Omp25+ L7/L12 were immunized in mice and isolation of serum samples was done from tail veins on day 42. Estimation of IgG isotype levels in serum of immunized mice was done through ELISA. The antibodies used for ELISA were horseradish peroxidase (HRP) conjugated anti-mouse IgG1, IgG2a, and IgG2b antibodies and data is plotted as (mean (OD_450_ ± SD)).

In order to predict the Th1/Th2 bias of immune response, the relative IgG isotypes levels (IgG1, IgG2a and IgG2b) were analyzed in Omp25+L7/L12 immunized mice along with mice immunized solely with L7/L12 and Omp25. In the case of anti-L7/L12 antibodies, IgG1 levels were found to be significantly higher than IgG2a levels in the divalent vaccine as well as L7/L12 only immunized mice group, indicating a Th2 biased immune response in both (Figure 3a). Similarly, in the case of anti-Omp25 antibody levels, IgG1 levels were found to be higher than IgG2a (IgG1/IgG2a = 1.86), suggesting a Th2 biased immune response in divalent vaccine immunized mice. Interestingly, levels of IgG2a and IgG2b antibodies in Omp25+L7/L12 immunized mice were noteworthy (Figure 3b), predicting an elicitation of Th1 immune response in divalent vaccine immunized mice as well.

### 2.4. Evaluation of Protective Efficacy Conferred by Divalent Vaccine Candidate against B. abortus 544 Challenge

The protective efficacy of the Omp25+L7/L12 vaccine candidate along with groups immunized solely with Omp25 and L7/L12 was analyzed against *B. abortus* 544 infection. Two weeks after last immunization, the immunized mice were challenged with virulent *B. abortus* 544 through intraperitoneal route. The mice were sacrificed four weeks post-infection, and bacterial colony forming units (CFU) were determined. As shown in Table 1, the level of log_10_ CFU per spleen at 28 days post-challenge with *B. abortus* 544 was (4.820 ± 0.18) in Omp25+L7/L12 immunized mice. Consecutively, log_10_ protection conferred by the Omp25+L7/L12 group was 1.98 at 28 day post-challenge as compared to (PBS + alum) immunized mice indicating that the Omp25+L7/L12 vaccine candidate was effective at eliminating pathogenic *B. abortus* 544 from a mice model. Mice immunized with Omp25 and L7/L12 alone exhibited log_10_ units of protection as 1.46 and 1.75, respectively at 28 days post-challenge with *B. abortus* 544 as compared to (PBS + alum) immunized mice. Overall, upon analyzing the levels of protection of the divalent vaccine candidate against *B. abortus* 544 challenge, it was found that Omp25+L7/L12 immunized mice exhibited efficacious log_10_ units of protection against *B. abortus* 544 challenge along with its individual components, however S19 exhibited the maximum.

## 3. Discussion

Brucellosis is still a major public health concern and endemic in many countries, mainly in the Mediterranean region, eastern and western Africa, and parts of South and Central America. There is a substantial requirement to control and eradicate this disease caused by the *Brucella* genus [4,5,7,35,36]. The current vaccines in use, Strain 19 and RB51 despite being popular are still far from ideal [12,37]. They prevent infection in animal but offer potential risks such as attenuation reversal and virulence in susceptible hosts. In this context, subunit vaccines are better options as compared to live attenuated vaccines, since they are safe and do not revert back to pathogenic strain upon administration [13,14,15]. Further, recombinant subunit vaccines protect against a given pathogen by activating humoral and cellular arms of immunity based upon a specific antigen along with used adjuvant, which makes them competent and useful in the vaccine field. There are certain proteins in *Brucella* species which can provide significant protection against the disease and are conserved throughout, such as L7/L12 [17], Omp19, Omp31 [18], BLS [19], BP26 [15], and Omp25 [20,21]. Among these, *omp25* and *l7/l12* genes in *Brucella* species encode for Omp25 and L7/L12 immunodominant proteins respectively, and have the potential to stimulate a strong humoral immune response along with providing protection against *Brucella* infections in mice models [15,17,20,28]. Earlier studies have suggested that outer membrane proteins from *Brucella* such as Omp25 [38], Omp10 [39], Omp19 [39], BP26 [40], Omp28 [41,42], and Omp31 [30] can distinguish between *Brucella*-infected animals and non-infected ones in an efficient and accurate way, withdrawing the false positive results in the field due to cross-reacting antibodies. Further multivalent subunit vaccine formulations possess the capability to generate a wide range of immunogens that may result in better protection than their univalent counterpart [24,25,26]. In this study, we evaluated humoral immune response and protective efficacy of a divalent vaccine candidate consisting of rOmp25 and rL7/L12 against *Brucella* infection in mice. The foremost point to be explored in this study was whether two components combined together in a divalent vaccine have the capability to show a synergistic response and promote a heightened immune response, or if some kind of competitive inhibition occurs among them? Aluminum hydroxide (Alum) is beneficial since it is inexpensive and has been certified as the safest adjuvant for use by the United States Food and Drug Administration [31,32,43]. Alum creates a depot effect at the site of injection, resulting in a slow release of adsorbed antigens and an elevation in the immune response. The intraperitoneal route of administration was chosen because it helps to quickly absorb antigens into the vasculature, which leads to a rise of antigen drainage into the spleen and activation of immune cells circulating in the lymph nodes [44].

The results in this study exhibited that humoral immune response was elevated in mice immunized with the divalent vaccine Omp25+L7/L12 as compared to the control group (PBS + alum). The divalent vaccine (Omp25+L7/L12) was found immunogenic with high IgG levels against both of its components, rOmp25 and rL7/L12 (Figure 2), depicting that divalent vaccine has the potential to exhibit synergy among its individual components and elevate the immune response against virulent *Brucella abortus* 544 challenge. During bacterial infection, Th2-mediated immune response is characterized by synthesis and increase in the level of IgG1 antibodies [45,46] whereas Th1 immune response is represented by levels of IgG2a antibody along with IFN-γ cytokine levels. The IgG subclass (IgG1, IgG2a and IgG2b) detection in Omp25+L7/L12 immunized mice exhibited that IgG1 levels were significantly higher as compared to IgG2a levels, predicting a more prominent Th2 immune response in case of anti-Omp25 and anti-L7/L12 antibodies (Figure 3). Although individual vaccinated group generated antibodies specifically to single antigen immunized whereas divalent vaccine candidate resulted in generation of antibodies against both antigens, rOmp25 and rL7/L12. Further, immunization with alum as an adjuvant has also been suggested to enhance antibody response in mice [43,47]. It helps in enhancement of antigen uptake and presentation to antigen-presenting cells, which results in promotion of Th2 immune responses [47,48]. Therefore, it is possible that alum has helped to increase antibody titer and elevate the humoral immune response in divalent vaccine immunized mice [49]. The analysis of protective efficacy in different mice groups after infection with virulent *Brucella abortus* 544 showed that Omp25+L7/L12 immunized mice exhibited a significant increase in log_10_ protection (1.98) as compared to the control (i.e. alum immunized mice) at 28 days after challenge (*p* value < 0.001; Table 1). This specifies that Omp25+L7/L12 immunized mice were capable of eliminating pathogenic *B. abortus* 544 compared to the control group. On comparing log_10_ units of protection at 28 days after challenge in individual protein immunized mice, rOmp25 (1.46) and rL7/12 (1.75) with alum as the adjuvant, it was observed that Omp25+L7/L12 immunized mice showed a superior level of protection against *B. abortus* 544 infection, however S19 exhibited the maximum. 

It is noteworthy that although *B. abortus* recombinant subunit vaccines show very promising results in mice models, the immune responses recognized in mice models may not reflect the protection achieved in natural hosts such as cattle after immunization [11]. Therefore, further studies determining protective efficacy in other animal models such as rats, guinea pigs, and monkeys are also encouraged before proceeding towards cattle administration [50]. Recombinant vaccines also need multiple booster administrations along with adjuvants and a combination of several antigens, which makes them economically unsuitable for cattle immunization [51]. Hence, there is a need to decrease the production cost, search for effective and affordable adjuvants, and reduce the expense of recombinant protein purification in order to make these vaccines economical for mass administration. 

In a nutshell, this preliminary study shows that the combination of rOmp25 and rL7/L12 elicited steady immune responses against both antigens in mice. Further, when mice were immunized with the Omp25+L7/L12 vaccine candidate, a significant reduction in *B. abortus* 544 load in mice spleens was observed, implying the use of divalent vaccine (Omp25+L7/L12) as an improved vaccine candidate against brucellosis. Nevertheless, this study illustrates the potential of a divalent vaccine in providing host immunity and protection against *B. abortus* challenge, suggesting the use of a divalent recombinant vaccine candidate as an advanced approach in the future against brucellosis.

## 4. Materials and Methods

### 4.1. Plasmids and Bacterial Strains

*E. coli* DH5α was used for propagation of recombinant plasmids. *E. coli* BL21 (DE3) and C43 strains were used for expression of rL7/L12 and rOmp25 proteins, respectively. *E. coli* strains were cultured using Luria–Bertani (LB) medium. Kanamycin was added to the medium at a final concentration of 50 µg/mL. *B. abortus* 544 and S19 strains were obtained from the Indian Veterinary Research Institute, Bareilly, India. *Brucella abortus* 544 was cultured in tryptic soy medium. Experiments involving *B. abortus* 544 and S19 strains were performed in a biosafety Level 3 laboratory at Jawaharlal Nehru University (JNU), Delhi, India. 

### 4.2. Expression and Purification of Recombinant Proteins

For formulation of the divalent vaccine, Omp25 and L7/L12 antigens of *Brucella abortus* were PCR amplified using gene specific primers and cloned in pET28(a) vector (Table 2). The expression of proteins was done in *E. coli*. To purify rOmp25, recombinants were grown in terrific broth until OD_600_ ~ 0.5–0.6, and then induction was done using 1 mM IPTG for 5 h at 37 °C. Further purification of rOmp25 was done from the insoluble inclusion bodies fraction, using the urea-denaturing method and on-column refolding [20]. To purify rL7/L12, recombinants were grown in LB medium containing kanamycin to OD_600_ ~ 0.7–0.8 followed by induction using 1 mM IPTG for 5 h at 37 °C. Both the proteins were affinity purified using immobilized nickel-nitrilotriacetic acid (Ni-NTA) agarose columns equilibrated in PB buffer (100 mM potassium phosphate buffer, pH 8.0) and eluted using 100–500 mM imidazole in PB. Purified proteins were analyzed by SDS-PAGE for content and purity. The dialysis of purified proteins was done against phosphate buffer saline (pH 7.4).

### 4.3. Immunoblotting

For immunoblotting, the recombinant proteins were resolved by 12% SDS-PAGE followed by electroblotting onto nitrocellulose membrane. The membrane was further blocked using 3% BSA followed by incubation with anti-Omp25+L7/L12 antibody (1:5000 dilution, raised in mice) for 1 h. After subsequent washing, binding specificity was checked using AP-conjugated goat anti-mouse IgG antibody (Catalog no. sc-2047, Santa Cruz Biotechnology, USA) [52,53,54].

### 4.4. Immunization of Respective Proteins in Mice

Four to six week old female BALB/c mice (inbred) were obtained from the National Centre for Laboratory Animal Sciences, Hyderabad, India. Recommendations from the Institutional Animal Ethics and Biosafety Committee were regularly followed during mouse experiments. In brief, mice were caged under sterile conditions in micro-isolators, fed with pathogen-free food and water *ad libitum* during consecutive immunizations. Once infected with *B. abortus* 544, mice were maintained at the BSL-3 animal facility of JNU for evaluation of protective efficacy.

For immunization of rL7/L12 and rOmp25, the optimized dose of each antigen was considered as mentioned in earlier reports [1,20]. Briefly, mice were grouped and immunized through the intraperitoneal route, either with Omp25 (30 μg) or L7/L12 (40 μg) alone or in combination as a divalent vaccine candidate with alum as an adjuvant. Two boosters were administered at regular intervals of 2 weeks, and 1X PBS with alum and *B. abortus* S19 immunized mice groups were taken as controls. For prime immunization and subsequent booster immunization, 100 µl emulsion of the required antigen and alum in 1X PBS was injected in each mouse. The blood was collected from each mouse on day 0, 14, 28, and 42 from tail veins and sera was extracted through centrifugation at 15,600 *g* for 20 min, followed by storage at −80 °C for further analysis.

### 4.5. Elucidation of End-Point Antibody Titer

An enzyme-linked immunosorbent assay (ELISA) was used to analyze serum antibody titer. In brief, 96-well microtiter plates (NuncMaxiSorp) were coated overnight with 500 ng/well of capture antigen (rOmp25 or rL7/L12) in PBS at 4 °C. The plates were washed three times using PBST (PBS with 0.1% tween 20) followed by blocking using 2% BSA in PBS for 2 h at 37 °C. The antibody titer in the sera of respective antigen immunized mice along with the divalent vaccine immunized mouse group was assessed by priming dilutions of the same, in triplicates, at 37 °C for 1 h. Washing of the plates was done using PBST followed by addition of horseradish peroxidase (HRP)-conjugated anti-mouse secondary antibodies (Catalog no. sc-2005, Santa Cruz Biotechnology, USA) at 1:10,000 dilution for 1 h at 37 °C [53,54]. The plates were further incubated with OptEIA TMB substrate (BD Biosciences, USA) for calorimetric assay and the reaction was stopped using 1N HCl. Absorbance of the plate was measured at 450 nm through Tecan’s Sunrise absorbance microplate reader. End point titer was evaluated as the reciprocal of highest dilution giving absorbance greater than the threshold value. Threshold value was calculated as the mean of absorbance plus three times standard deviation of 1:1000 dilution of the control group (PBS + alum).

### 4.6. Analysis of IgG Isotypes in Immunized Mice

The IgG isotypes (IgG1, IgG2a and IgG2b) were detected in immunized mice using ELISA as described above. For secondary antibodies, anti-mouse IgG1-HRP (Catalog no. sc-2060), anti-mouse IgG2a-HRP (Catalog no. sc-2061) and anti-mouse IgG2b-HRP conjugated antibodies (Catalog no. sc-2062) (raised in goat; Santa Cruz Biotechnology, USA) were used and absorbance at 450 nm was measured [53].

### 4.7. Evaluation of Protective Efficacy of Vaccine Candidate

Two weeks after the final booster immunization (day 42), mice groups immunized with PBS, rOmp25, rL7/L12, and divalent vaccine candidate (rOmp25+rL7/L12) were challenged with 2 × 10^5^ cells of *B. abortus* 544 through the intraperitoneal route. *B.abortus* S19 was injected on day 0 in respective group, and challenge was done after 21 days with 2 × 10^5^ cells of virulent *B. abortus* 544. After 4 weeks of infection, mice from each group were euthanized through cervical dislocation. Their spleen was extracted under sterile conditions and finally homogenized in PBS using probe homogenizer. For CFU count, various dilutions of the spleen homogenate were prepared and plated on tryptic soya agar followed by incubation at 37 °C for 48 h in the presence of 5% CO_2_. Total splenic load was calculated and represented as Log_10_ CFU mean ± standard deviation (SD). Log_10_ units of protection were determined by calculating the difference between the log_10_ CFU of PBS injected group (control) and vaccinated group.

### 4.8. Statistical Analysis

The results are represented as mean ± SD and are reported as data of three different sets of experiments. The statistical significance in antibody titer was calculated using two-tailed Student’s t-test. (* represents *P* < 0.05; ** represents *P* < 0.01; *** represents *P* < 0.001, **** represents *P* < 0.0001).

**Ethical statement:** All mice experiments were performed while abiding by the rules of Institutional Animal Ethics Committee (IAEC), Jawaharlal Nehru University, New Delhi, India guidelines. All experiments involving virulent *Brucella abortus* 544 and *Brucella abortus* S19 strain have been performed in Biosafety level-3 (BSL-3) facility.

## Figures and Tables

**Table 1:** Bacterial proliferation in the spleen of mice immunized with rOmp25, rL7/L12, divalent vaccine candidate (Omp25+L7/L12) and control, using alum as adjuvant. The mice were infected with *B. abortus* 544 through intraperitoneal route and the splenic bacterial load was determined by plating dilutions of the splenocytes suspension on the tryptic soya agar plates followed by incubation at 37 °C in the presence of 5% CO_2_ for 48 h. Data is represented as mean ± S.D.

S. No	Vaccine Candidate	Log_10_ Spleen Bacillary Load at Day 28 Post-Challenge (Log_10_ CFU)	Log_10_ Units of Protection at Day 28 Post-Challenge
1.	rOmp25	5.338 ± 0.75	1.46
2.	rL7/L12	5.05 ± 0.27	1.75
3.	Omp25+L7/L12	4.820 ± 0.18	1.98
4.	PBS	6.80 ± 0.58	–
5.	*B. abortus* S19	4.21 ± 0.31	2.59

**Table 2:** Description of strains used in this study.

S. No.	Protein Name	Strain Used for Purification of Protein	Reference
1.	rOmp25	*omp25* gene was cloned in pET28a at BamHI and SalI sites and expressed in *E. coli* C43 cells.	Goel et al. 2012 [20]
2.	rL7/L12	L7/L12 ribosomal gene was cloned in pET28a at NcoI and XhoI sites and expressed in *E. coli* BL21(DE3) cells.	Singh et al. 2015 [1]
